# Interspecific synchrony of seed rain shapes rodent‐mediated indirect seed–seed interactions of sympatric tree species in a subtropical forest

**DOI:** 10.1111/ele.13405

**Published:** 2019-10-20

**Authors:** Xifu Yang, Chuan Yan, Haifeng Gu, Zhibin Zhang

**Affiliations:** ^1^ State Key Laboratory of Integrated Management of Pest Insects and Rodents in Agriculture Institute of Zoology Chinese Academy of Sciences Beijing 100101 China; ^2^ CAS Center for Excellence in Biotic Interactions University of Chinese Academy of Sciences Beijing 100049 China

**Keywords:** Apparent competition, apparent mutualism, apparent predation, masting, rodents, scatter‐hoarding, seed dispersal effectiveness, seed rain, seed traits

## Abstract

Animal‐mediated indirect interactions play a significant role in maintaining the biodiversity of plant communities. Less known is whether interspecific synchrony of seed rain can alter the indirect interactions of sympatric tree species. We assessed the seed dispersal success by tracking the fates of 21 600 tagged seeds from six paired sympatric tree species in both monospecific and mixed plots across 4 successive years in a subtropical forest. We found that apparent mutualism was associated with the interspecific synchrony of seed rain both seasonally and yearly, whereas apparent competition or apparent predation was associated with interspecific asynchrony of seed rain either seasonally or yearly. We did not find consistent associations of indirect interactions with seed traits. Our study suggests that the interspecific synchrony of seed rain plays a key role in the formation of animal‐mediated indirect interactions, which, in turn, may alter the seasonal or yearly seed rain schedules of sympatric tree species.

## Introduction

Apparent competition and apparent mutualism among sympatric tree species are two major predator‐mediated indirect interactions (Abrams *et al.* 1998; Veech 2000). Recently, apparent predation among seed species was also revealed as a category of indirect interaction (Lichti *et al.* 2014). Apparent competition exists when an increase in the seed abundance of one species leads to a second species experiencing higher predation because of the presence of a third species (such as a seed predator) in a patch, whereas apparent mutualism occurs when an increase in the seed abundance of the first species leads to an increase in the second species due to the presence of a third species (such as a seed disperser) (Veech 2000; Kitzberger *et al.* 2007; Xiao & Zhang 2016). Apparent predation exists when scatter‐hoarders perceive one species to be more useful for hoarding, and its presence may lead hoarders to alter their interactions with other species that are less valued for hoarding (Lichti *et al.* 2014; Pesendorfer & Koenig 2017).

Many studies have shown that there is apparent competition among sympatric tree species through their seeds by predator‐mediated interactions (e.g. Veech 2000; Dangremond *et al.* 2010; Norghauer & Newbery 2011). A few studies revealed that apparent mutualism (Kitzberger *et al.* 2007; Xiao & Zhang 2016) and apparent predation (Lichti *et al.* 2014; Pesendorfer & Koenig 2017; Bogdziewicz *et al.* 2018b) occur among tree species through seeds by rodents and birds. Moreover, recent studies showed that seed predation and dispersal of a given tree species can be indirectly affected by other sympatric tree species at the neighbourhood scale (Yi *et al.* 2011; Garzon‐Lopez *et al.* 2015; Yi & Wang 2015). However, strict assessments of the seed–seed interactions under animal predation and dispersal for a given tree species within or without a neighbourhood have been largely ignored (but see: Albrecht *et al.* 2015; Xiao & Zhang 2016; Bogdziewicz *et al.* 2018b).

Seed traits (e.g. seed size, nutrient quality, physical and chemical defences) vary greatly among different tree species (Jansen *et al.* 2004; Xiao *et al.* 2006b; Koenig *et al.* 2009; Wang & Chen 2009) and may play a vital role in determining animal‐mediated seed dispersal (Lai *et al.* 2014; Cao *et al.* 2016; Zhang *et al.* 2016a). In addition, sympatric tree species often produce seeds with contrasting traits that are either attractive or defensive to seed vectors (Jansen *et al.* 2004; Vander Wall 2010; Wang *et al.* 2016; Zhang *et al.* 2016b). In general, frugivores and granivores are often generalists; therefore, harvesting seeds of a given tree species may be influenced not only by its own seed traits but also by those of other sympatric tree species (Shimada 2001). In seed communities, indirect interactions may arise because seeds vary in size and palatability to animals (Emerson *et al.* 2012; Ostoja *et al.* 2013) or due to differences in the perishability of sympatric seed species (Lichti *et al.* 2014; Xiao & Zhang 2016). Although the identification of certain seed traits by seed predators has been shown to cause indirect seed–seed interactions among sympatric tree species (Lichti *et al.* 2014; Yi & Wang 2015; Xiao & Zhang 2016), most of these studies used only a few paired seed species in evaluating the indirect interactions (Garzon‐Lopez *et al.* 2015; Xiao & Zhang 2016; Bogdziewicz *et al.* 2018b; Yang *et al.* 2019).

Masting (or mast seeding), which synchronously produces large seed crops at irregular interannual intervals, is commonly seen in many perennial plant species in nature (Ims 1990; Kelly 1994; Kelly & Sork 2002), particularly for those species that are dispersed by food‐hoarding animals in temperate and tropical forests (Sork 1993; Jansen *et al.* 2004; Vander Wall 2010; Yang *et al.* 2019). Masting has been thought to have evolved under several selective pressures, for example, improved pollination success (Kelly *et al.* 2001; Pearse *et al.* 2015), enhanced seed dispersal mediated by animals (Vander Wall 2002; Pesendorfer *et al.* 2016) and increased predator satiation (Silvertown 1980; Fletcher *et al.* 2010; Bogdziewicz *et al.* 2018a). In contrast, masting may cause predator satiation (Jansen *et al.* 2004; Xiao *et al.* 2013) or increase seed dispersal (Vander Wall 2002; Li & Zhang 2007; Zwolak *et al.* 2016). Although masting has been found to be beneficial to forest regeneration of the focal species at both the population level (Li & Zhang 2007) and the individual level (Zhang *et al.* 2008), little is known of how the interspecific synchrony of masting schedules affects rodent‐mediated indirect seed–seed interactions (e.g. apparent competition or mutualism) of sympatric tree species.

The purpose of this study aimed to evaluate rodent‐mediated indirect interactions among sympatric tree species and to examine key factors (e.g. similarity of seed traits and interspecific synchrony of seed rain) affecting seed–seed interactions of sympatric tree species. We emphasise the testing the two hypotheses: the seed‐trait similarity hypothesis and the seed rain synchrony hypothesis in terms of seed dispersal. The seed‐trait similarity hypothesis suggests that similar seed traits would facilitate apparent competition because they have similar attractions to rodents for seed dispersal; otherwise, dissimilar seed traits would facilitate apparent predation because they present different attractions to rodents. The seed rain synchrony hypothesis suggests that the interspecific synchrony of seed rain would facilitate both seasonally and yearly seed dispersal and survival of both seed species (apparent mutualism), as suggested by the predator satiation hypothesis or by the predator dispersal hypothesis; otherwise, interspecific asynchrony of seed rain either seasonally or yearly would facilitate apparent competition or predation. Interspecific synchrony of seed rain of sympatric tree species would amplify the effects of masting effect of a single tree species, and then promote seed dispersal or survival of interacted sympatric tree species, resulting in an apparent mutualism between tree species. But asynchrony of masting of tree species would cause distinct effects on these tree species, leading to apparent competition or predation between them. This is because masting of one tree species would enhance its own seed dispersal by attracting animals, but would depress seed dispersal of the non‐masting tree species.

We tracked the seed fates of six paired sympatric seeds presented to rodents in both monospecific and mixed plots in the subtropical forest ecosystems of the Dujiangyan Region, Sichuan Province, southwestern China. We had the following predictions: (1) the higher the similarity of seed traits, the more likely it was that the paired plant species would show apparent competition as measured by seed scatter‐hoarding or seed dispersal effectiveness under dispersal by rodents, otherwise, (2) the paired plant species would show apparent predation; (3) the higher the interspecific synchrony of seed rain both seasonally and yearly, the more likely it was that the paired plant species would show rodent‐mediated apparent mutualism, otherwise, (4) the paired plant species would show rodent‐mediated apparent competition or predation in seed dispersal. Our results demonstrate that the interspecific synchrony of seed rain rather than similarity of seed traits shapes the rodent‐mediated indirect seed–seed interactions of sympatric tree species in a subtropical forest.

## Methods

### Study site and species

The field study was conducted in the Dujiangyan Region (altitude 600–1000 m, 31°04′ N–31°05′ N, 103°42′ E–103°43′ E), which is located in the transition zone between the Qinghai‐Tibetan Plateau and the plains of Chengdu, southwestern China. The annual mean temperature is 15.2 °C, and the annual precipitation is 1200–l800 mm. The weather is often cloudy and foggy, with 800–1000 annual mean hours of sunshine and an annual mean relative humidity of 80%. The habitat is a subtropical evergreen broad‐leaved forest, and the region is a hotspot of biodiversity in China. Vegetation is dominated by the Fagaceae species *Quercus serrata*, *Quercus variabilis*, *Cyclobalanopsis glauca*, *Castanopsis fargesii*, *Castanopsis ceratacantha*, *Lithocarpus hancei*, *Lithocarpus megalophyllus*, and the Anacardiaceae species *Choerospondias axillaris* and *Toxicodendron vernicifluum*. The shrubby understory is diverse and rich in the Theaceae species *Camellia sp* and in the Symplocaceae species *Symplocos sp.* Seed rains of these Fagaceae, Anacardiaceae, Theaceae and Symplocaceae species varied greatly across seasons and years, and their seeds are consumed and/or hoarded by several rodent species (Chang & Zhang 2014). *Apodemus draco*, *Apodemus chevrieri*, *Apodemus latronum*, *Niviventer fulvescens*, *Niviventer confucianus*, *Leopoldamys edwardsi* and *Micromys minutus* are the main rodent species (Yang *et al.* 2014; and this study). These rodents are common seed‐consuming and/or hoarding rodents in the area (Xiao *et al.* 2013). No diurnal rodents were found in the study region.

## Experimental design

### Seed selection

We selected six paired trees for monospecific and mixed seed placement by considering the differences in synchrony of seasonal and yearly seed rain, similarity of seed traits, and seed availability within the forests. *Q. serrata*, *Q. variabilis*, *C. glauca* and *C. fargesii* belong to the same family of Fagaceae and have similar seed traits; but their seed traits are very different from those of tree species of the Theacae (*C. oleigera*) or Anacardiaceae (*C. axillaris*) (Xiao *et al.* 2006b; Chang & Zhang 2014). Besides, these tree species also differ in seed rain across seasons and years (Xiao *et al.* 2005; Xiao *et al.* 2006b; Li *et al.* 2012), but quantitative analysis on the difference between them has not been conducted. We calculated the correlation coefficients of seasonal seed rain among these sympatric tree species (Table S1) using the seed rain data of these tree species we collected in 2014 before the tests (Fig. S1), to show the synchrony or asynchrony of seed rains across seasons. If the correlation was significant, we treated them as synchronous pairs; if not significant, we treated them an asynchronous pairs (Table 1). The yearly synchrony of seeds among these tree species is likely driven by climate or other factors, but we were not able to determine the synchrony of yearly seed rain of these tree species before the tests due to lack of yearly seed rain data. Seed rains often vary across seasons and years; thus, the synchrony of seasonal and yearly seed rain among sympatric tree species used for statistical analysis of this study was calculated based on the observed data we collected during 2015–2018 (Tables S2 and S3). The seed traits were measured based on seeds we collected in 2016 (Table S4).

**Table 1:** The experimental information for paired seeds of six sympatric tree species during 2015–2018. In each year, three to four paired seeds were tested in three replicated patches, by considering different synchrony of seasonal rain and seed traits. Each paired seeds was repeated for 1–4 years, depending on the seed availability. *Q. serrata*, *Q. variabilis* and *C. oleifera* were used more frequently due to their abundant seed rains

Groups	Seed species	Number of patches (replicates)				Patch code	No. of released seeds	Seasonal synchrony of seed rain Observed from 2015 to 2018 [that based on data in 2014]	Seed trait similarity Observed from 2015 to 2018 [that based on previous studies]
		2015	2016	2017	2018				
Paired 1	Qv + Co	3	3	3	3	B12, F, H	5760	No[no]	No[no]
Paired 2	Qs + Cg			3	3	B21, K, M	2880	Yes[yes]	Yes[yes]
Paired 3	Qv + Qs		3	3	3	B11, D, A	4320	Yes[yes]	Yes[yes]
Paired 4	Co + Cg		3	3	3	B22, L, C	4320	Yes[yes]	No[no]
Paired 5	Co + Qs	3				B11, L, A	1440	Yes[yes]	No[no]
Paired 6	Ca + Cf	3			3	B22, D, M	2880	No[no]	No[no]

Qv, *Q. variabilis*; Ca, *C. axillaris*; Co, *C. oleifera*; Qs, *Q. serrata*; Cg, *C. glauca*; and Cf, *C. fargesii*. ‘Yes’ or ‘No’ indicate the observed synchrony of seasonal seed rain or similarity of seed traits between paired seeds during 2015–2018. ‘yes’ or ‘no’ indicates the assumed synchrony of seed rain before tests based on correlation results in Table S1 using seed rain data collected in 2014, or assumed seed similarity based our previous studies (Xiao *et al.* 2006b; Chang & Zhang 2014).

### Seed dispersal experiment

We conducted all field trials from early November to mid‐January for four successive years (2015–2018). A total of 21 600 intact seeds were randomly selected from all plant seeds for conducting seed dispersal experiments. A 0.3–0.4 mm diameter hole was drilled through the husk near the germinal disc of each seed using a portable electrical drill. The seeds were tied with small, light red plastic tags (3.6 × 2.5 cm, < 0.1 g) through the hole using a 10 cm long thin steel wire; each tag was coded with a serial number using a marker pen to identify every seed (see: Xiao *et al.* 2006a; Yang *et al.* 2018). When small rodents buried the tagged seeds in the soil, the tags were often left on the surface, making them easy to visually locate. Seed tagging has been shown to have a negligible effect on seed removal and caching by small rodents (Xiao *et al.* 2006a).

**Figure 1:**
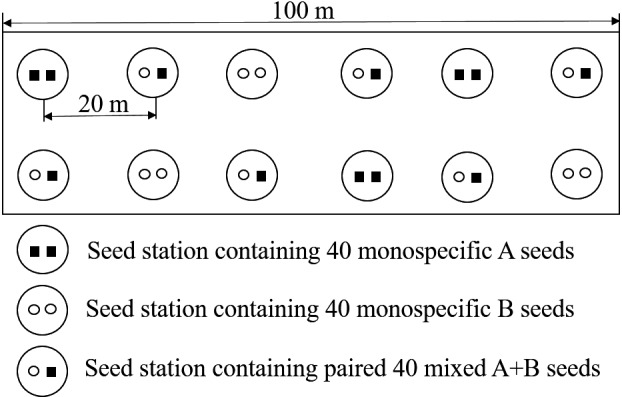
Scheme for the placement of seeds with monospecific and mixed seed stations in each patch. A and B represent seeds of two different sympatric tree species in our study area.

To explore whether the presence of other given seeds (e.g. seed B) showed different influences on the seed dispersal of the given seeds (e.g. seed A), we established 12 seed stations (plots), spaced 20 m apart in each patch, as described by Xiao & Zhang (2016). Each seed station included 40 seeds; half of all seed stations contained only 40 monospecific seeds (three stations contained seed A and three stations contained seed B); the other half contained 40 mixed seeds (seed A and seed B), and monospecific and mixed stations were interlaced; each patch included total 480 seeds (Fig. 1).

After seed release, we assessed the tagged seeds every 2 days for the first 8 days, and then appropriately extended the time between assessments according to the seed dispersal rates until most seeds had been removed by small rodents. At the same time, we randomly searched a 25 m radius around each station with equal effort (2–3 h by two people for each patch) and recorded the fate of the tagged seeds. The released seed fates in each station were defined as follows: intact *in situ*; eaten *in situ*; eaten after removal; scatter‐hoarded after removal (buried in the surface soil or beneath the leaf litter); missing (not located due to a visual barrier or more likely being larder‐hoarded in burrows or tree cavities). We also recorded the dispersal distances of the cached seeds from their source seed stations. Cached seeds were marked using a numbered bamboo stick to allow easy relocation. During subsequent visits, we searched for cached seeds until they were recovered (eaten or removed) by animals. If a marked cache was removed, the area around the cache was extensively searched in an attempt to relocate the seeds. Seed cache survival and seedling establishment from scatter hoards were surveyed in April and/or May of the next year. The seedlings were identified by checking the numbers on the plastic tags attached to the remnant cotyledons of seed seedlings. We repeated the same procedures in 2016, 2017 and 2018 with different acorn crop sizes. Seed fates, such as intact *in situ*, eaten *in situ*, eaten after removal and missing and dispersal distances were only recorded but not analysed in this paper.

Under rodent dispersal and predation, seeds are eaten, scatter‐hoarded or larder‐hoarded. Eaten seeds or larder‐hoarded seeds impose negative effects on seedling establishment of trees. Only scatter‐hoarded seeds are able to become established into seedlings if they finally escaped from predation by rodents. The proportion of scatter‐hoarded seeds is a key factor in affecting plant regeneration (Jansen *et al.* 2004). Higher proportions of scatter‐hoarded seeds represent a more mutualistic interaction between rodents and trees (Chang & Zhang 2014; Wang *et al.* 2014; Zhang *et al.* 2015). Thus, proportion of scatter‐hoarded seeds (Xiao & Zhang 2016) or proportion of seedling establishment (Cao *et al.* 2016) is often used for measuring the mutualistic relation between tree and rodents.

In this study, we used both scatter‐hoarded seeds (SH) and seed dispersal effectiveness (SDE) to test the indirect seed–seed interactions between trees by comparing their differences under the treatment of monospecific and mixed seeds. By following Xiao & Zhang (2016), SH was measured as follows: (the number of seeds scatter‐hoarded by rodents)/(the number of seeds released at the seed stations) × 100%. According to Lichti *et al. *(2014), SDE was measured as follows: (the number of seeds removed by rodents/the number of seeds released at the seed stations) × (the number of seeds overwinter survival given removal/the number of seeds removed by rodents) × 100% = (the number of survived seeds after overwintering)/(the number of released seeds) × 100%. There are some differences between of SH and SDE. SH does not measure the final survival of the seeds, whereas SDE measures the final survival by the next spring. To cross‐validate our results, we used both indices in this study.

### Seed rain dynamics

To determine the interspecific synchrony of seed rain for both the seasonal and yearly domains, we measured the seed fall using seed traps following Yang *et al. *(2018). Each trap sampled a 1 × 1 m^2^ area. In the middle of August 2015, we set up 171 seed traps that were suspended 0.8 m above the ground using bamboo or trunk posts in 13 patches (for details, see Yang *et al.* 2018). Considering the different sizes of the plots, three to seven seed traps were placed in a plot, in two or four sampling lines with a spacing of 10 m between adjacent traps. During 2015–2018, we collected fallen seeds for approximately 2 weeks from the middle of August to late December when the seeds matured. The seed crop was measured as the mean number of seeds per square meter (No./m^2^). We divided seeds collected from seed traps into three categories: intact, infested and aborted seeds. Intact seeds are defined as follows: the pulp is easily to peel, the seeds are full, earth yellow or brown, the colour is bright and the seed coat surface is smooth without evidence of infection by larva of insects (Xiao *et al.* 2001). In this study, because intact seeds were more responsive to seed yield dynamics, we only analysed the number of intact seeds that likely germinated after dispersal by animals and provided effective food resources for animals.

### Rodent population changes

We monitored rodent populations in 13 patches where we conducted the seed release experiments from October to November from 2015 to 2018. In each patch, we set a 4 × 10 trapping grid with an interval of 10 m and used wire live traps (30 × 13 × 12 cm) baited with fresh chestnuts to trap small rodents for five consecutive nights (200 trap nights). Traps were placed at 15:00–18:00 h in the afternoon and were checked at 7:00–9:00 h in the next morning. The captured rodents were weighed and identified to determine species, sex and reproductive status, marked (individual rodents were marked using coloured paint to permit identification during the survey), and released immediately *in situ*. Detailed methods are described in Yang *et al. *(2018).

In this study, 638 individuals from 10 rodent species were captured from 2015 to 2018, and the trap success varied from 0.08% to 5.96% in the 13 patches among four years. *A. draco, N. confucianus, N. fulvescens, L. edwardsi* and *A. chevrieri* were the most abundant species in the experimental plot. In 2017, there were more trap successes relative to the other 3 years (all *P* < 0.001, Chi‐square tests).

### Statistical analyses

We used proportion of scatter‐hoarded seeds and seed dispersal effectiveness to evaluate rodent‐mediated indirect seed–seed interactions for sympatric tree species. The data of scatter‐hoarded seeds represent the binary outcome of seed fates: if a seed was scatter‐hoarded by rodents, it was assigned to 1; otherwise, it was assigned to 0. By following Xiao & Zhang (2016), we analysed the seed fate with binary data (0,1) using generalised linear mixed models (GLMMs) with a binomial distribution and logit‐link function to test the significant effects of neighbourhood treatment (monospecific vs. mixed seeds) as a fixed factor on proportion of scatter‐hoarded seeds, and with year and patch as the random factors. Due to the low rate of overwinter survival given dispersal during a year or for a patch, we pooled the data from year and patch, and significant differences of seed dispersal effectiveness between treatments were tested using Chi‐square tests if the theoretical value was at least 5, otherwise, using Fisher’s exact tests. R software (R Core Team 2018) was used for the statistical analysis of the GLMMs (Package lme4; Bates *et al.* 2015) and Chi‐square tests (or Fisher’s exact tests).

Pearson correlation was used to calculate the seed‐trait similarity and interspecific seasonal or yearly seed rain synchrony among six sympatric seed species. The monthly seed rain was calculated by averaging the seed rain (No./m^2^) of each patch, all patches and all years in order, respectively. First, we calculated the seed rain of each patch by averaging the seed rain of all seed traps of each month for a given year. Second, we calculated the seed rain of each month for a given year by averaging the seed rain of all patches. Finally, we calculated the monthly seed rain of a given month during the study period by averaging the monthly seed rain of all years, and we calculated the yearly seed rain by averaging all monthly seed rain during the study period. The synchrony of seasonal seed rain was calculated based on the correlation coefficient of the monthly seed rain between every two tree species. The synchrony of yearly seed rain was calculated as the correlation coefficient of yearly seed rain between every two tree species. Factor analysis was used to identify the key principal components in determining the seed traits of the six tree species (Fig. S3). The data of seed traits and seed crops were log_10_‐transformed to meet the normal distribution assumptions of the statistical models. SPSS statistics (version 20) were used for these statistical analysis. All statistical tests were two‐tailed, and the α level was set at 0.05.

## Results

### Effects of neighbourhood treatment on scatter‐hoarding and seed dispersal effectiveness

**Figure 2:**
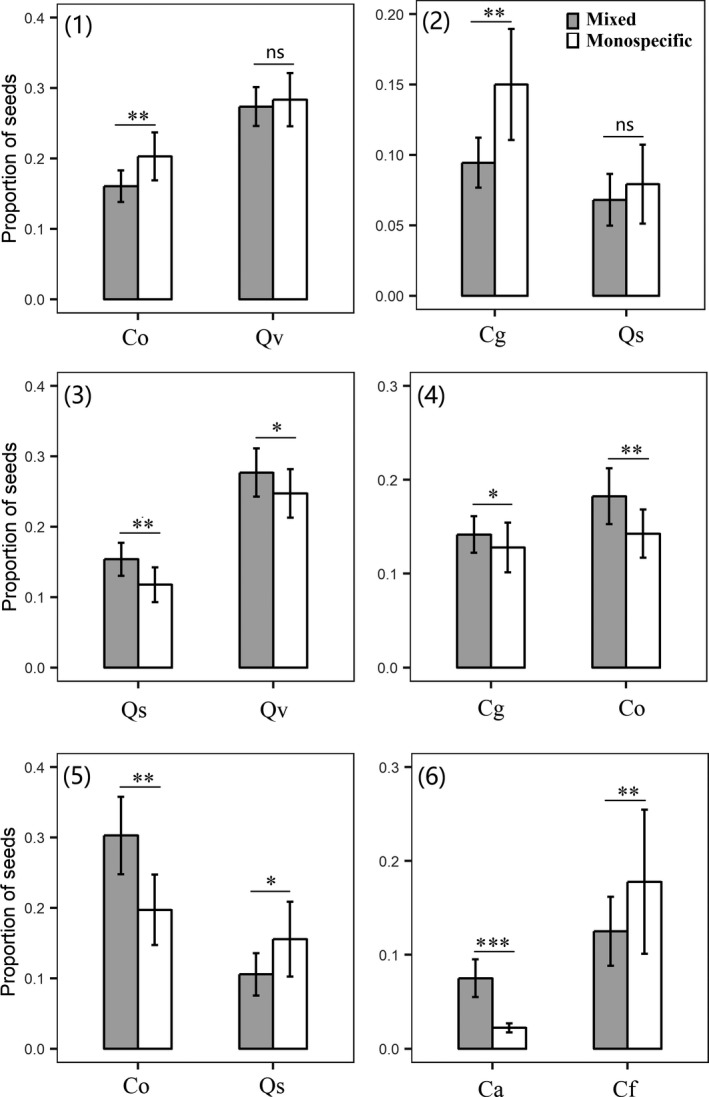
Proportions of scatter‐hoarding of seeds by sympatric rodent dispersers in monospecific and mixed stations. Qv, *Q. variabilis*; Ca, *C. axillaris*; Co, *C. oleifera*; Qs, *Q. serrata*; Cg, *C. glauca*; and Cf, *C. fargesii*. Neighbourhood treatments: monospecific, monospecific stations; mixed, mixed stations. Qv and Co (1), Cf and Qs (2), Qs and Qv (3), Cg and Co (4), Co and Qs (5) and Ca and Cf (6). Error bars show the standard error (1 SE) (*P < 0.05; **P < 0.01; ***P < 0.001).

**Figure 3:**
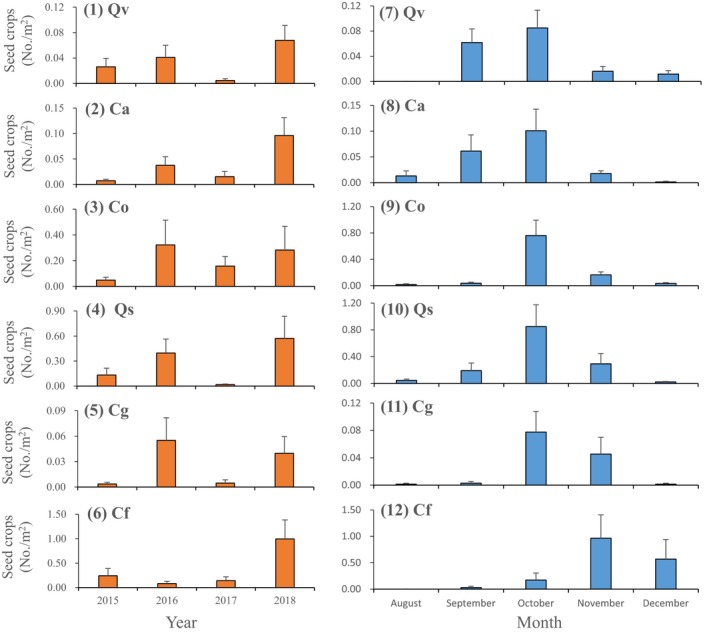
Seed rain dynamics at yearly (1–6) and at seasonal (7–12) levels for six sympatric seed species from 2015 to 2018. Qv, *Q. variabilis*; Ca, *C. axillaris*; Co, *C. oleifera*; Qs, *Q. serrata*; Cg, *C. glauca*; and Cf, *C. fargesii*. Data are expressed as the mean ± SE.

The scatter‐hoarding and seed dispersal effectiveness were strongly and consistently affected by the neighbourhood among six pairs of seeds. The presence of neighbouring seeds for the paired seeds of *Q. variabilis* and *C. oleifera* (Paired 1) and for the paired seeds of *Q. serrata* and *C. glauca* (Paired 2) showed significantly or marginally negative effects on the scatter‐hoarding and seed dispersal effectiveness of given seeds, indicating apparent competition (Fig. 2, (1) and (2); Fig. S2; Table 2). The presence of neighbouring seeds for the paired seeds of *Q. variabilis* and *Q. serrata* (Paired 3) and for the paired seeds of *C. oleifera* and *C. glauca* (Paired 4) showed significant positive effects on the scatter‐hoarding and seed dispersal effectiveness of the given seeds, indicating apparent mutualism (Fig. 2, (3) and (4); Fig. S2; Table 2). The presence of neighbouring seeds for the paired seeds of *Q. serrata* and *C. oleifera* (Paired 5) and for the paired seeds of *C. axillaris* and *C. fargesii* (Paired 6) showed significant positive or negative effects on the scatter‐hoarding and seed dispersal effectiveness of the given seeds, indicating apparent predation (Fig. 2, (5) and (6); Fig. S2; Table 2).

**Table 2:** Summary of the indirect effects of the presence of neighbouring seeds on the scatter‐hoarding and seed dispersal effectiveness of the given seeds based on generalised linear mixed models and Chi‐square tests (or Fisher’s exact tests)

Groups	Seed species	Fixed factors	Scatter‐hoarding†, †				Seed dispersal effectiveness‡, ‡		Total effects§, §
			Estimate	SD	*Z*	*P*	x^2^	*P*	
Paired 1	*Quercus variabilis*	Neighbour	−0.092	0.087	−1.049	0.294	0.956	1.000	Apparent competition
	*Camellia oleifera*	Neighbour	−0.328	0.100	−2.280	0.001**, **	3.913	0.048*, *	
Paired 2	*Quercus serrata*	Neighbour	−0.170	0.205	−0.829	0.407	0.166^¶^	0.125	Apparent competition
	*Cyclobalanopsis glauca*	Neighbour	−0.538	0.166	−3.235	0.001**, **	2.588	0.043*, *	
Paired 3	*Quercus variabilis*	Neighbour	0.199	0.097	2.044	0.041*, *	13.211	<0.001***, ***	Apparent mutualism
	*Quercus serrata*	Neighbour	0.343	0.125	2.733	0.006**, **	10.352	0.001**, **	
Paired 4	*Camellia oleifera*	Neighbour	0.342	0.120	2.856	0.004**, **	9.317	0.002**, **	Apparent mutualism
	*Cyclobalanopsis glauca*	Neighbour	0.264	0.125	2.114	0.035*, *	5.155	0.023*, *	
Paired 5	*Quercus serrata*	Neighbour	−0.456	0.227	−2.011	0.044*, *	–	–	Apparent predation
	*Camellia oleifera*	Neighbour	0.573	0.176	3.262	0.001**, **	–	–	
Paired 6	*Choerospondias axillaris*	Neighbour	1.114	0.259	4.305	<0.001***, ***	20.636	<0.001***, ***	Apparent predation
	*Castanopsis fargesii*	Neighbour	−0.466	0.159	−2.923	0.003**, **	33.727	<0.001***, ***	

Neighbour represents neighbourhood treatment;

* *P* < 0.05;

** *P* < 0.01;

*** *P* < 0.001.

^†^ Statistical results based on generalised linear mixed models;

^‡^ Statistical results based on Chi‐square tests (or Fisher’s exact tests ¶);

^§^ Total effects based on ^†^and/or ^‡^Statistical results; – Indicates no detectable.

### Effects of seed traits

Pearson correlation analysis indicated that the acorn traits of *Q. variabilis*, *Q. serrata*, *C. glauca* and *C. fargesii* were positively correlated with each other (all *P* < 0.05); the seed traits of *C. axillaris* were positively associated with *C. oleifera* seed traits (*P* < 0.05; Table S5). Similar to the results of Fig. S3, using Principal Components Analysis (PCA), two factors were extracted from these seed traits. These two factors explained 94.879% of the variance in seed traits. Factor 1 represents acorns, including the seeds of *Q. variabilis*, *Q. serrata*, *C. glauca* and *C. fargesii*; Factor 2 represents other seeds including the seeds of *C. axillaris and C. oleifera* (Fig. S3). We did not find any obvious association of seed‐trait similarity with the seed–seed indirect interactions (Table 3).

**Table 3:** Comprehensive statistical results of seed traits, synchrony of seed rain and rodent‐mediated indirect interactions from six pairs of sympatric seed species

Groups	Seed species	Correlation coefficient by seed traits†, †	Correlation coefficient by yearly seed crops‡, ‡	Correlation coefficient by seasonal seed crops§, §	Total effects¶
Paired 1	*Quercus variabilis*	−0.159	0.612	0.745	Apparent competition
	*Camellia oleifera*				
Paired 2	*Quercus serrata*	0.999***, ***	0.840	0.940**, **	Apparent competition
	*Cyclobalanopsis glauca*				
Paired 3	*Quercus variabilis*	0.875**, **	0.977§, §	0.814‡, ‡	Apparent mutualism
	*Quercus serrata*				
Paired 4	*Camellia oleifera*	−0.187	0.935‡, ‡	0.925‡, ‡	Apparent mutualism
	*Cyclobalanopsis glauca*				
Paired 5	*Quercus serrata*	−0.179	0.766	0.979§, §	Apparent predation
	*Camellia oleifera*				
Paired 6	*Choerospondias axillaris*	−0.270	0.858‡, ‡	−0.447	Apparent predation
	*Castanopsis fargesii*				

* *P* < 0.05;

** *P* < 0.01;

*** *P* < 0.001.

^†^ Results from Table S5;

^‡^ Results from Table S2;

^§^ Results from Table S3;

^¶^ Results from Table 2.

### Effects of interspecific synchrony of seed rain

At the yearly level, correlation analysis indicated that the seed crops of *Q. variabilis* were positively correlated with the seed crops of *Q. serrata* over 4 years (*P* < 0.01); the seed crops of *C. oleifera* were positively associated with the seed crops of *C. glauca* over 4 years (*P* < 0.05) and the seed crops of *C. axillaris* were positively correlated with the seed crops of *C. fargesii* over 4 years (*P* < 0.05) (Tables 3 and S2). The seed rain of *Q. variabilis* and *Q. serrata* (Paired 3), *C. oleifera* and *C. glauca* (Paired 4), *C. axillaris* and *C. fargesii* (Paired 6) showed higher synchrony over the years, whereas the other pairs showed lower synchrony of seed rain across years (Fig. 3; Tables 3 and S2).

At the seasonal level, correlation analysis indicated that the seed crops of *Q. serrata* were positively correlated with the seed crops of *Q. variabilis* (*P* < 0.05), *C. glauca*, and *C. oleifera* (all *P* < 0.01); the seed crops of *C. glauca* were positively correlated with the seed crops of *C. oleifera* (*P* < 0.05) (Tables 3 and S3). The seasonal seed rain between *Q. serrata* and *C. glauca* (Paired 2), *Q. variabilis* and *Q. serrata* (Paired 3), *C. oleifera* and *C. glauca* (Paired 4), and *Q. serrata* and *C. oleifera* (Paired 5) showed higher synchrony, except for those between *Q. variabilis* and *C. oleifera* (Paired 1) and *C. axillaris* and *C. fargesii* (Paired 6) (Fig. 3; Tables 3 and S3).

We found that two sets of paired seeds (Paired 3 and 4) showed rodent‐mediated apparent mutualism in seed dispersal only when their seed rains exhibited higher synchrony both seasonally and yearly (Table 3). Otherwise, paired seeds showed rodent‐mediated apparent competition (Paired 1 and 2) or apparent predation (Paired 5 and 6) in seed dispersal when their seed rains had lower synchrony either seasonally or/and yearly (Table 3).

## Discussion

Our study revealed three types of animal‐mediated indirect seed–seed interactions, including apparent competition, apparent mutualism and apparent predation. We found that apparent mutualism was associated with the interspecific synchrony of seed rain both seasonally and yearly, and that apparent competition or apparent predation was associated with asynchrony of seed rain either seasonally or yearly, support the prediction by seed rain synchrony hypothesis. Seed‐trait similarity was not associated with rodent‐mediated indirect seed–seed interactions as predicted by the seed‐trait similarity hypothesis. Our study provided insight into the ecological and evolutionary mechanisms of animal‐mediated seed–seed indirect interactions and the synchrony of seed rain of sympatric tree species.

Seed fate is critical for achieving plant population regeneration during seed dispersal (Vander Wall 2001; Jansen *et al.* 2004; Zhang *et al.* 2017). In general, the more seeds that are scatter‐hoarded by seed hoarders, the better the seed germination and the establishment of seedlings are (Wang *et al.* 2014; Zhang *et al.* 2016a). The seed dispersal effectiveness was ideally measured as the dispersal activities of a disperser (Schupp 1993; Schupp *et al.* 2010). However, in many studies, there is often a small proportion of seeds that become established as seedlings in fields. Therefore, scatter‐hoarding was also used as an indicator to evaluate the benefits of seed dispersal (e.g. Vander Wall 2002; Li & Zhang 2007; Fletcher *et al.* 2010; Xiao & Zhang 2016). In this study, rodents removed 64.21% of the seeds (*n* = 13 870) from the seed stations and scatter‐hoarded 17.62% (*n* = 3806) of the seeds. Finally, 407 (1.88%) of the seeds survived overwinter, and only 127 (0.59%) of these seeds survived to the stage of taproot establishment (including seedling establishment). We used both scatter‐hoarding and seed dispersal effectiveness in this study, and the results were similar.

### Animal‐mediated indirect seed–seed interactions

Previous studies have provided solid evidence that sympatric tree species have the potential to enhance or reduce the overall seed mortality patterns through seed–seed interaction mediated via shared seed predators (Yi *et al.* 2011; Garzon‐Lopez *et al.* 2015; Bogdziewicz *et al.* 2018a). However, most of these studies used only a few paired seed species for evaluating the indirect interactions (Garzon‐Lopez *et al.* 2015; Xiao & Zhang 2016; Bogdziewicz *et al.* 2018b; Yang *et al.* 2019). By using six paired seed species and tracking 21 600 tagged seeds, we found that there were three animal‐mediated indirect seed–seed interactions (Figs. 2 and S2; Tables 2 and 3). The paired seeds of *Q. variabilis* and *C. oleifera* (Paired 1) and the paired seeds of *Q. serrata* and *C. glauca* (Paired 2) showed apparent competition, supporting previous observations (Abrams *et al.* 1998; Veech 2000; Dangremond *et al.* 2010; Xiao & Zhang 2016). We found that the paired seeds of *Q. variabilis* and *Q. serrata* (Paired 3) and the paired seeds of *C. oleifera* and *C. glauca* (Paired 4) showed apparent mutualism, supporting previous findings (Kitzberger *et al.* 2007; Garzon‐Lopez *et al.* 2015; Xiao & Zhang 2016; Yang *et al.* 2019). The paired seeds of *Q. serrata* and *C. oleifera* (Paired 5) and the paired seeds of *C. axillaris* and *C. fargesii* (Paired 6) showed apparent predation, supporting previous studies (Lichti *et al.* 2014; Pesendorfer & Koenig 2017; Bogdziewicz *et al.* 2018b). These indirect interactions between sympatric tree species may change the quality of the dispersal services provided by animals to their plant partners (Bogdziewicz *et al.* 2018b).

### Effects of seed traits

A few studies suggest that rodent‐mediated indirect interaction among sympatric tree species are largely affected by contrasting seed traits and species abundance (Yi & Wang 2015; Xiao & Zhang 2016; Yang *et al.* 2019), which may affect the coexistence of tree species (Garzon‐Lopez *et al.* 2015). These studies have the limitation of small sample sizes of species pairs for evaluating the effects of seed traits (such as tannin concentrations and germination schedules) on indirect interaction between seeds of sympatric tree species mediated by rodents (Lichti *et al.* 2014; Xiao & Zhang 2016). In this study, we selected six paired seeds with different seed traits (Fig. S3; Tables S4 and S5). We did not find clear associations of seed‐trait similarities on the seed–seed indirect interactions; therefore, the seed‐trait similarity hypothesis and our Predictions 1 and 2 were not supported. This result was likely because a trade‐off exists between different seed characteristics, resulting from the combined effects of multiple features, such as nutrient contents and physical and chemical defences (Zhang & Zhang 2008; Vander Wall 2010; Zhang *et al.* 2015; Zhang *et al.* 2016b). More works are needed to identify the effects of seed traits in affecting the seed–seed indirect interactions.

### Effects of interspecific synchrony of seed rain

Sympatric tree species bearing seeds usually exhibit synchronised masting years (Kelly 1994; Kelly & Sork 2002; Koenig & Knops 2005), but the underlying mechanism has not been fully elucidated. Masting of sympatric tree species has been proposed to improve their own seed dispersal, resulting in mutualism (Vander Wall 2002; Jansen *et al.* 2004; Li & Zhang 2007), but this hypothesis has been rarely tested at the community level. When two or more masting species coexist, seed predators and/or dispersers may show different functional responses to resource types when compared to the case of a single masting species, relying on context‐dependent indirect seed–seed interactions (Lichti *et al.* 2014; Yi & Wang 2015; Pesendorfer *et al.* 2016; Sawaya *et al.* 2018; Yang *et al.* 2019). To the best of our knowledge, there is no study assessing the association between the indirect seed–seed interaction and synchrony of seed rain using large datasets. Our results showed that the interspecific synchrony of seed rain both seasonally and yearly was associated with rodent‐mediated apparent mutualism between sympatric tree species; otherwise, sympatric tree species showed rodent‐mediated apparent competition or predation when the asynchrony of seed rain occurred either seasonally or yearly, supporting our seed rain synchrony hypothesis and our Predictions 3 and 4.

## Conclusion

In summary, our study revealed that the interspecific synchrony of seed rain rather than the similarity of seed traits was associated with the rodent‐mediated indirect seed–seed interactions of sympatric tree species. In theory, rodent‐mediated indirect seed–seed interactions may further alter the masting schedules of the sympatric tree species and then promote the coexistence of sympatric tree species. Future studies should be directed towards identifying the ecological and evolutional significance of the observed associations.

## Authorship

ZZ, YC and YX designed the study; YX and GH collected the field data; YX and YC did the analyses; YX and ZZ wrote the first draft of the manuscript; and all authors contributed intellectually to the manuscript.

## Data Availability

Data available from the Figshare Repository: https://doi.org/10.6084/m9.figshare.9896078.
