# Supplementary material for: Interspecific synchrony of seed rain shapes rodent‐mediated indirect seed–seed interactions of sympatric tree species in a subtropical forest
Source: Ecol Lett. 2019 Oct 20;23(1):45–54. doi: 10.1111/ele.13405 (PMC6916184; doi:10.1111/ele.13405)
Supplement: Supplementary file 1 [file ELE-23-45-s001.docx]

**Supporting information**

Interspecific synchrony of seed rain shapes rodent-mediated indirect seed-seed interactions of sympatric tree species in a subtropical forest

Xifu Yang, Chuan Yan, Haifeng Gu, Zhibin Zhang^*^

^*^Corresponding author, Email: zhangzb@ioz.ac.cn (+86-01064807069)

**Table S1** Pearson correlation coefficients of seasonal seed rain between sympatric tree species in 2014.

**Table S2** Pearson correlation coefficients of yearly seed rain between sympatric tree species during 2015 to 2018.

**Table S3** Pearson correlation coefficients of seasonal seed rain between sympatric tree species during 2015 to 2018.

**Table S4** Morphological, nutrient compositions and caloric value for six seed species.

**Table S5** Pearson correlation coefficients of seed traits between sympatric tree species.

**Figure S1** Seed fall dynamics and crop size of six sympatric seed species in 2014.

**Figure S2** Seed dispersal effectiveness under rodent predation and dispersal in monospecific and mixed stations.

**Figure S3** Principal components of seed traits of six sympatric tree species.

**Table S1** Pearson correlation coefficients of seasonal seed rain between sympatric tree species in 2014. *, *P*<0.05; **, *P*<0.01; ***, *P*<0.001.

|  | *Quercus variabilis* | *Choerospondias axillaris* | *Camellia oleifera* | *Quercus serrata* | *Cyclobalanopsis glauca* |
| --- | --- | --- | --- | --- | --- |
| *Choerospondias axillaris* | 1.000*** |  |  |  |  |
| *Camellia oleifera* | 0.621 | 0.621 |  |  |  |
| *Quercus serrata* | 0.759* | 0.759* | 0.933*** |  |  |
| *Cyclobalanopsis glauca* | 0.389 | 0.389 | 0.950*** | 0.802* |  |
| *Castanopsis fargesii* | -0.088 | -0.088 | 0.497 | 0.240 | 0.559 |

**Table S2** Pearson correlation coefficients of yearly seed rain between sympatric tree species during 2015 to 2018. *, *P*<0.05; **, *P*<0.01.

|  | *Quercus variabilis* | *Choerospondias axillaris* | *Camellia oleifera* | *Quercus serrata* | *Cyclobalanopsis glauca* |
| --- | --- | --- | --- | --- | --- |
| *Choerospondias axillaris* | 0.897* |  |  |  |  |
| *Camellia oleifera* | 0.612 | 0.691 |  |  |  |
| *Quercus serrata* | 0.977** | 0.906* | 0.766 |  |  |
| *Cyclobalanopsis glauca* | 0.717 | 0.633 | 0.935* | 0.840 |  |
| *Castanopsis fargesii* | 0.750 | 0.858* | 0.223 | 0.657 | 0.171 |

**Table S3** Pearson correlation coefficients of seasonal seed rain between sympatric tree species during 2015 to 2018. *, *P*<0.05; **, *P*<0.01.

|  | *Quercus variabilis* | *Choerospondias axillaris* | *Camellia oleifera* | *Quercus serrata* | *Cyclobalanopsis glauca* |
| --- | --- | --- | --- | --- | --- |
| *Choerospondias axillaris* | 0.971** |  |  |  |  |
| *Camellia oleifera* | 0.745 | 0.812* |  |  |  |
| *Quercus serrata* | 0.814* | 0.872* | 0.979** |  |  |
| *Cyclobalanopsis glauca* | 0.599 | 0.666 | 0.925* | 0.940** |  |
| *Castanopsis fargesii* | -0.364 | -0.447 | -0.079 | -0.071 | 0.233 |

**Table S4** Morphological, nutrient compositions and caloric value for six seed species. Bold numbers show the largest values of the seed traits.

| Seed traits | *Quercus variabilis* | *Choerospondias axillaris* | *Camellia oleifera* | *Quercus serrata* | *Cyclobalanopsis glauca* | *Castanopsis fargesii* |
| --- | --- | --- | --- | --- | --- | --- |
| Seed weight (g) | **5.35 ± 0.84** | 1.77 ± 0.32 | 1.41 ± 0.61 | 1.46 ± 0.30 | 0.90 ± 0.16 | 0.59 ± 0.09 |
| Seed coat thickness (mm) | 0.56 ± 0.09 | **1.59 ± 0.30** | 0.33 ± 0.09 | 0.39 ± 0.07 | 0.31 ± 0.06 | 0.39 ± 0.07 |
| Crude protein (%) | 4.43 | **23.77** | 8.63 | 4.16 | 3.69 | 3.13 |
| Crude fat (%) | 2.54 | 46.48 | **52.92** | 2.56 | 2.35 | 0.15 |
| Crude starch (%) | 49.50 | 0.92 | 1.87 | 42.19 | 50.42 | **72.75** |
| Crude fiber (%) | 2.08 | **14.86** | 3.32 | 2.18 | 2.02 | 1.61 |
| Tannin (%) | **6.69** | 0.12 | 0.10 | 4.81 | 6.08 | 0.04 |
| Caloric value (KJ/g) | 10.62 | **24.88** | 22.96 | 9.34 | 10.57 | 13.39 |
| Caloric value of per seed (KJ) | **32.02** | 4.15 | 13.00 | 6.16 | 5.88 | 4.51 |

Seed morphological traits, Mean ± S.D., *N*=50. Seed chemical analysis is conducted in duplicate for a mixture of intact seeds for each tree species, and seed nutrient compositions are measured by the Measure Center of Grain Quality, Ministry of Agriculture, China. Dry-kernel caloric values are calculated by the average gross-energy equivalents of protein (17.2 KJ /g), fat (38.9 KJ /g) and carbohydrates (17.2 KJ /g) (see similar analysis in Zhang & Zhang 2008).

Reference

Zhang, H. & Zhang, Z. (2008). Endocarp thickness affects seed removal speed by small rodents in a warm-temperate broad-leafed deciduous forest, China. *Acta Oecol.*, 34, 285-293.

**Table S5** Pearson correlation coefficients of seed traits between sympatric tree species. *, *P*<0.05; **, *P*<0.01; ***, *P*<0.001.

|  | *Quercus variabilis* | *Choerospondias axillaris* | *Camellia oleifera* | *Quercus serrata* | *Cyclobalanopsis glauca* |
| --- | --- | --- | --- | --- | --- |
| *Choerospondias axillaris* | -0.372 |  |  |  |  |
| *Camellia oleifera* | -0.159 | 0.890** |  |  |  |
| *Quercus serrata* | 0.875** | -0.269 | -0.179 |  |  |
| *Cyclobalanopsis glauca* | 0.865** | -0.278 | -0.187 | 0.999*** |  |
| *Castanopsis fargesii* | 0.851** | -0.270 | -0.194 | 0.994*** | 0.994*** |

**
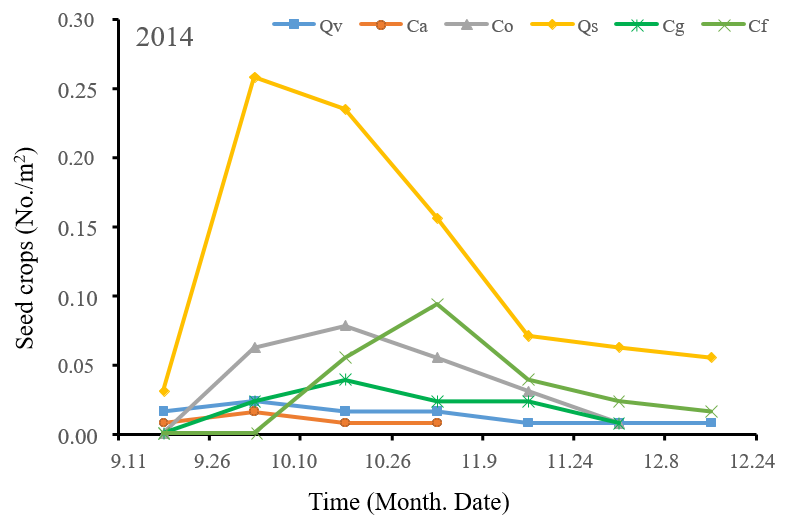
**

**Figure S1** Seed fall dynamics and crop size of six sympatric seed species in 2014. Qv, *Quercus variabilis*, Ca, *Choerospondias axillaris*, Co, *Camellia oleifera*, Qs, *Quercus serrata*, Cg, *Cyclobalanopsis glauca*, Cf, *Castanopsis fargesii*.

**
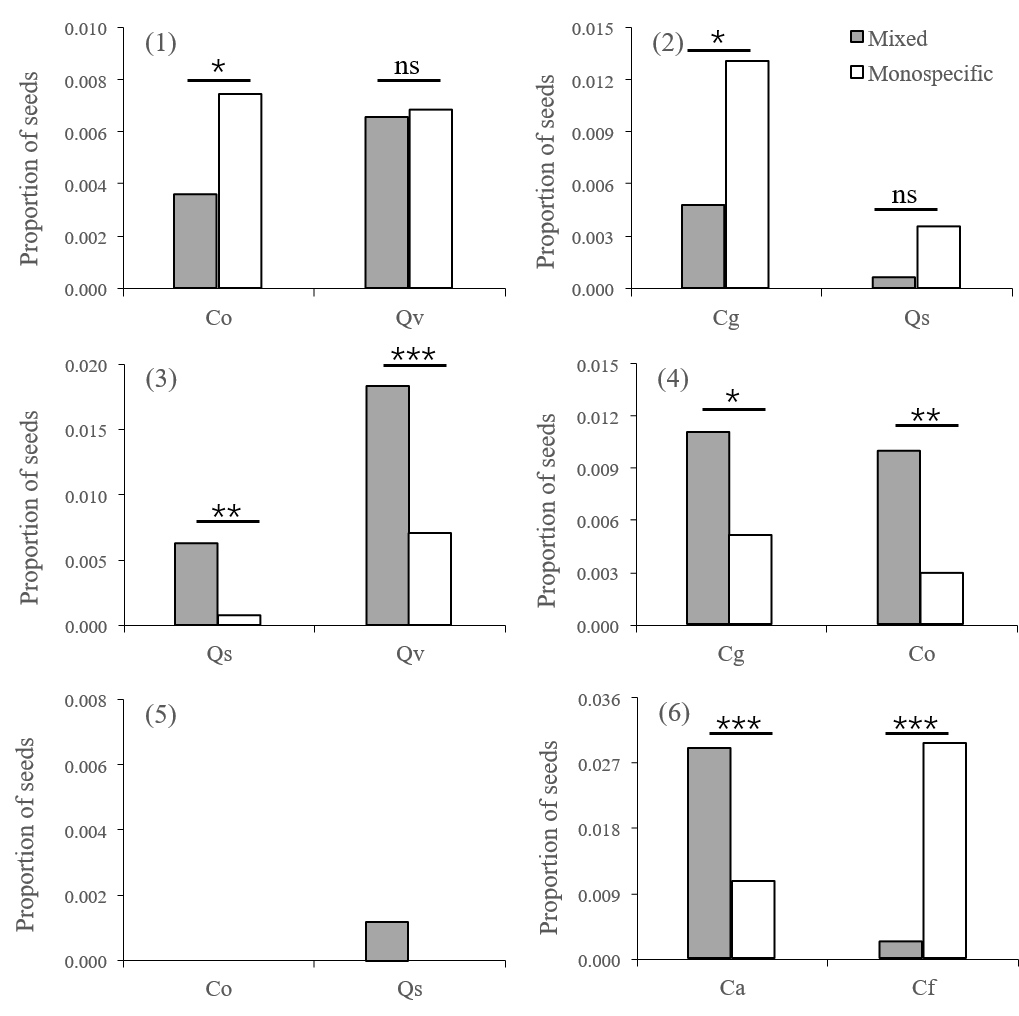
**

**Figure S2** Seed dispersal effectiveness under rodent predation and dispersal in monospecific and mixed stations. *Quercus variabilis* (Qv), *Camellia oleifera* (Co), *Quercus serrata* (Qs), *Cyclobalanopsis glauca* (Cg), *Choerospondias axillaris* (Ca), and *Castanopsis fargesii* (Cf). Neighborhood treatments: monospecific, monospecific stations; mixed, mixed stations. Qv and Co (1), Cf and Qs (2), Qs and Qv (3), Cg and Co (4), Co and Qs (5), and Ca and Cf (6). (*, *P*<0.05; **, *P*<0.01; ***, *P*<0.001).


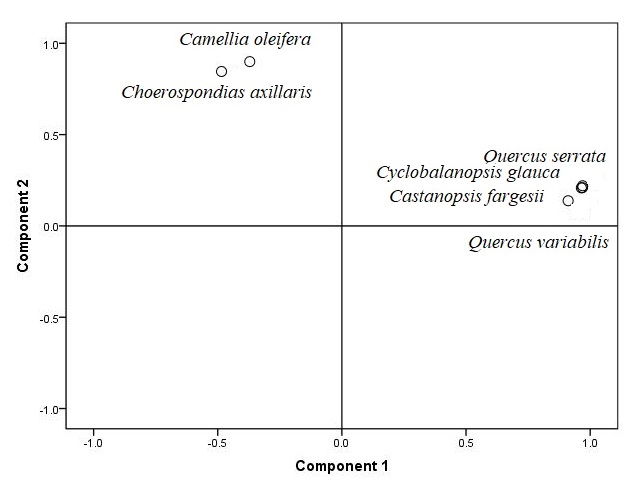


**Figure S3** Principal components of seed traits of six sympatric tree species. Cumulative variance of principle component 1 and 2 (eigenvalue > 1, vortexed) is 94.879%.
